# Microarray-Based Cancer Prediction Using Soft Computing Approach

**DOI:** 10.4137/cin.s2655

**Published:** 2009-05-26

**Authors:** Xiaosheng Wang, Osamu Gotoh

**Affiliations:** 1 Department of Intelligence Science and Technology, Graduate School of Informatics, Kyoto University, Kyoto 606–8501, Japan; 2 National Institute of Advanced Industrial Science and Technology, Computational Biology Research Center, Tokyo 135-0064, Japan

**Keywords:** gene expression profiles, cancer prediction, soft computing, rough set theory, feature selection, decision rules

## Abstract

One of the difficulties in using gene expression profiles to predict cancer is how to effectively select a few informative genes to construct accurate prediction models from thousands or ten thousands of genes. We screen highly discriminative genes and gene pairs to create simple prediction models involved in single genes or gene pairs on the basis of soft computing approach and rough set theory. Accurate cancerous prediction is obtained when we apply the simple prediction models for four cancerous gene expression datasets: CNS tumor, colon tumor, lung cancer and DLBCL. Some genes closely correlated with the pathogenesis of specific or general cancers are identified. In contrast with other models, our models are simple, effective and robust. Meanwhile, our models are interpretable for they are based on decision rules. Our results demonstrate that very simple models may perform well on cancerous molecular prediction and important gene markers of cancer can be detected if the gene selection approach is chosen reasonably.

## Introduction

Conventional tumor diagnostic methods based on the morphological appearance of tumors are not always effective as misdiagnoses often occur. On the other hand, a wide variety of studies have revealed cancer to be a disease involving dynamic changes in the genome. Therefore, using molecular markers of cancers might be an alternative approach to the diagnosis of tumors. The rapid advances in gene expression microarray technology that enable simultaneously measuring the expression levels for tens of thousands of genes in a single experiment, make the detection of cancerous molecular markers possible.^1^ Since the pioneering work of Golub et al in applying gene expression monitoring by DNA microarray to cancer classification,^2^ many investigations of using microarray technology to build cancer diagnosis, prognosis or prediction classifiers have been conducted. In general, the major difficulty in this topic is how to effectively identify the genes pertaining to the pathogenesis of specific cancers from the extremely high-dimensionality gene expression data, which often contain a large amount of noise caused by irrelevant genes. On the other hand, compared with the measured quantities of gene expression levels in experiments, the numbers of samples are severely limited. That often influences prediction accuracy. In this extreme of very few observations on very many features, it is natural and perhaps essential to investigate feature selection and regularization methods.^3^ Feature selection, i.e. gene filtering, is particularly crucial for microarray-based cancer prediction since the number of irrelevant genes for prediction may be huge, and as long as feature selection is performed reasonably, accurate prediction is achieved with even the simplest of predictive models.^4^

Various methods of building cancer predictors have been proposed such as Clustering, SVMs (Support Vector Machines), k-NNs (k-Nearest Neighbours), ANNs (Artificial Neural Networks), GAs (Genetic Algorithms), Naive Bayes (NB), DTs (Decision Trees), RSs (Rough Sets), EPs (Emerging Patterns), et al. In this article, we explore the use of rule-based pipelines to construct cancer predictors as the rule-based methods are more likely to be accepted by biologists and clinicians for they are easily understood. This kind of approaches like DTs,^5^ RSs,^6^ EPs^7^ etc. have been commonly utilized to produce cancer predictors by many investigators.^7^–^14^ In addition, we attempt to employ one or two genes to conduct cancer prediction. The same problem also has been addressed by some investigators.^15^,^16^

Our method is based on rough set theory, originally proposed by Pawlak in the early 1980s,^6^ which can be applied for analysis of both precise and imprecise data.^17^ In,^8^–^11^ rough set theory is applied for cancer classification and prediction. A majority of these studies conduct feature selection by the attribute reduction approach, one core idea of rough set theory. However, to our knowledge, rough sets attribute reductions are computationally expensive, and the resultant reducts maybe are not unique. Moreover, the reducts cannot ensure high prediction performance because there maybe exists redundancy between the attributes in one reduct.^18^ To avoid expensive cost in computing attribute reductions, we select the features (genes) with perfect *attribute depended degree*, a concept from rough set theory, and then create rule classifiers by the chosen genes instead of running attribute reductions. As it is very difficult to find the single genes or gene pairs with perfect attribute depended degree in terms of the canonical definition, we extend the concept of attribute depended degree to the more flexible soft computing framework. Using the extended definition of attribute depended degree, we can detect some single genes or gene pairs with indeed strong class discriminatory power while they will be ignored if the conventional attribute depended degree standard is employed. Consequently, although the rules derived from the detected genes or gene pairs might not be absolutely true, they are comparatively reliable and able to perform effective prediction.

We apply our algorithm to the four noted gene expression datasets: central nervous system (CNS) tumor, colon tumor, lung cancer, and diffuse large B-cell lymphoma (DLBCL). They are available from the Kent Ridge Bio-medical Data Set Repository (http://datam.i2r.a-star.edu.sg/datasets/krbd/). We validate the efficacy of our method by leave-one-out cross-validation (LOOCV), and compare our results with other already published research outcomes. Furthermore, we examine and analyze the biological relevance of the selected genes.

## Results

### CNS tumor dataset

In the dataset, we first try to find the single genes with high class discriminative power. When *α* is set to 0.9 or 0.85, there is no gene with *α* depended degree equal to 1 occurring in all the 60 training sets; when *α* is set to 0.8, gene U28963_at occurs in 59 out of the 60 training sets; when *α* is set to 0.75 and 0.7, there are two and six genes occurring in all the 60 training sets, respectively. In every training set, each of the six genes results to two decision rules, which are used to predict the test sample. The final prediction estimate is the average of 60 test results. Table 1 shows the prediction results by the six genes. Subsequently, we attempt to seek for the gene pairs with strong class discriminative ability. When *α* is set to 0.9, no gene pair is detected; when *α* is set to 0.85, only one gene pair is detected; when *α* is reduced to 0.8, eleven gene pairs are found. In general, each gene pair produces four decision rules. Then we apply the four decision rules to classify the test sample and the average of 60 test results is the prediction estimate of the gene pair. Table 2 shows the prediction results by the eleven gene pairs.

Here we denote the expression level of gene *G* by g(*G*). When the first sample is left out as the test set, and the remaining samples set is trained by the learning algorithm, the selected gene U28963_at will give rise to two decision rules:

- If g(U28963_at) ≤ 431, then Class 1;
- If g(U28963_at) >431, then Class 0.

The two rules have 81% and 84% confidence, respectively. One can use the two rules to classify the test set. When another sample instead of the first one is left out, gene U28963_at will result to two similar decision rules:

- If g(U28963_at) ≤ *x*, then Class 1;
- If g(U28963_at) > *x*, then Class 0.

*x* equals to 431 or is close to it. Anyway, the rules imply that if gene U28963_at is up-regulated in one CNS tumor patient, the patient will be more inclined to succumb to the disease. The other chosen genes give rise to similar form of rules.

Likewise, when the first sample is left out for test while the remaining samples are retained for training, the selected gene pair D83542_ at—S71824_at will generate four decision rules:

- if g(D83542_at) ≤ 280.5 and g(S71824_at) ≤ 434, then Class 1;
- if g(D83542_at) ≤ 280.5 and g(S71824_at) > 434, then Class 1;
- if g(D83542_at) > 280.5 and g(S71824_at) ≤ 434, then Class 1;
- if g(D83542_at) > 280.5 and g(S71824_at) > 434, then Class 0.

The four rules possess 100%, 100%, 89% and 88% confidence, respectively. They can be simplified into equivalent three rules:

- if g(D83542_at) ≤ 280.5 , then Class 1;
- if g(S71824_at) ≤ 434, then Class 1;
- if g(D83542_at) > 280.5 and g(S71824_at) > 434, then Class 0.

The three rules have 100%, 92% and 88% confidence, respectively. One can employ the four or alternative three rules to classify the test set. When another sample instead of the first one is left out, gene pair D83542_at—S71824_at will generate four similar decision rules. These rules indicate that if both D83542_at and S71824_at are highly expressed in one CNS tumor patient, then the patient will be very likely to succumb to the disease. Similar rules can be derived by the other chosen gene pairs.

### Colon tumor dataset

Using the same learning algorithm for the dataset, we screen the genes and gene pairs with comparatively high prediction performance. The results are presented in Table 3 and Table 4. As before, decision rules can be induced by the selected genes or gene pairs.

### Lung cancer dataset

In the dataset, when *α* is set to 0.8, no any gene is detected; when *α* equals to 0.75, eight genes are detected; when *α* is reduced to 0.7, no more genes are found. To make the decision rules induced by gene more reliable, we exclude the genes with missing values. When *α* is set to 0.9, 0.85 or 0.8, no any gene pair is found; when *α* is reduced to 0.75, eight gene pairs are detected. The results are presented in Table 5 and Table 6.

### DLBCL dataset

In the dataset, when *α* is set to 0.7, there are four genes selected; when *α* increases to 0.75, no any gene is found. With respect to gene pairs, when *α* is set to 0.9 or 0.85, no any gene pair is found; when *α* decreases to 0.8, there are 22 gene pairs chosen. The results are presented in Table 7 and Table 8. Table 8 shows only 20 out of the 22 gene pairs. The other two gene pairs are omitted because of their overly low prediction accuracy.

## Comparison of Prediction Performance

### CNS tumor dataset

The dataset is dataset C mentioned in^19^ that is used to analyze the outcome of the treatment for central nervous system embryonal tumor patients. In this dataset, we gain the 83% and 90% best prediction accuracy using one and two genes respectively. In,^19^ Pomeroy et al use a k-NNs algorithm to construct outcome predictor based on gene expression. The reported statistically significant gene size for k-NN models ranges from 2 to 21 genes, with the best prediction made by an 8-gene model that made 13/60 classification errors. Several other prediction algorithms including weighted voting, SVMs, and IBM SPLASH are also tested in.^19^ In,^20^ Zhang et al propose a hybrid approach, which combines discernibility matrix, the filter strategy and the wrapper method to select gene sets. Then they adopt the classifiers C4.5 and NaiveBayes to evaluate the prediction performance of the gene sets. Their prediction accuracy by LOOCV is 75% for C4.5 using 20 genes and 86.67% for Naive-Bayes using 29 genes. In,^12^ Tan et al use decision trees (Single C4.5, Bagging C4.5, AdaBoost C4.5) to perform prediction tasks on cancerous microarray data including the CNS tumor dataset. They first employ Fayyad and Irani’s^21^ discretization method to screen 74 genes for the actual learning process. Their highest prediction accuracy is 88% by tenfold cross-validation. The comparison of our methods with the others is summarized in Table 9. The table shows that our results are better than almost all the other compared results from previous studies.

#### Colon tumor dataset

The dataset is first studied by Alon et al.^22^ They propose two-way clustering approach that classify genes into functional groups and classify tissues based on their gene expression similarity. Since their original work, the dataset has been frequently investigated by other investigators. In this dataset, we reach the 84% and 90% highest prediction accuracy using one and two genes respectively. Table 10 compares the prediction results of our work with some other studies. The table demonstrates that whereas we use the least genes, our prediction accuracy is superior to or matches the others.

### Lung cancer dataset

In this dataset, we obtain the 85% and 82% highest prediction accuracy using one and two genes respectively. With respect to this dataset, we only find that Zhang et al report their study results^20^ apart from the original paper.^23^ Table 11 presents the comparison between our method and that provided in.^20^ Although their best prediction accuracy by the HFW feature selection approach is a little higher than ours, the numbers of the genes used by them far exceed ours. As for the other feature selection approaches including FCBF, CFS-SF and ReliefF, the prediction performance caused by them is inferior to ours.

### DLBCL dataset

In this dataset, we achieve the 78% and 90% best prediction accuracy using one and two genes respectively. Table 12 gives the comparison between our method and that provided in^20^ and^24^ Obviously, our results dominate the others.

## Analysis of Biological Relevance

### CNS tumor dataset

In this dataset, we identify six genes with comparatively high prediction performance individually. The six genes are U28963_at, X99050_rna1_at, D83542_at, S71824_at, U37673_ at, and D86974_at. According to the decision rules induced by the genes, we suspect that they are all over-expressed in the patients who succumb to their disease. As expected, three out of the six genes are picked as the markers of survival by Pomeroy et al.^19^ The three genes are referred to as GPS2 (U28963_at), beta-NAP (U37673_at) and KIAA0220 gene (D86974_at) respectively. Moreover, beta-NAP and KIAA0220 gene are the members of the 8-gene model by which k-NN makes optimal prediction. In addition, three genes named Human polyposis locus (DP1 gene), NSCL1 and VLCAD which compose the gene pairs with strong prediction power are also identified as markers of survival by Pomeroy et al.^19^

GPS2 encodes a protein involved in G protein-mitogen-activated protein kinase (MAPK) signaling cascades. The function of this gene may be signal repression. Zhang et al indicate that GPS2 interacts with another protein RFX4_v3 to modulate transactivation of genes involved in brain morphogenesis.^25^ Therefore, the dysregulation of GPS2 may be closely correlated with the pathogenesis of CNS tumor. Beta-NAP, a cerebellar degeneration antigen, is a neuron-specific vesicle coat protein.^26^ NSCL1 is the gene expressed predominantly in the developing nervous system.^27^ Our rules indicate that if the gene is over-expressed, the patients will be more likely to succumb to the CNS tumor. It coincides with the observation reported in.^27^

#### Colon tumor dataset

In this dataset, we identify 21 genes which can result to relatively efficient prediction individually. Some of these genes have been proved to tightly link with the pathogenesis of colon tumor or other tumors. Desmin is identified as one of three known hub cancer genes in colon cancer-specific gene network.^28^ Our rules indicate that the gene is down-regulated in colon tumor samples. The same conclusion is provided in.^29^ The gene CRP encodes a member of the cysteine-rich protein (CSRP) family. This gene family includes a group of LIM domain proteins, which may be involved in regulatory processes important for development and cellular differentiation. The LIM/double zincfinger motif found in this gene product occurs in proteins with critical functions in gene regulation, cell growth, and somatic differentiation. This gene has been reported to be associated with several cancers.^30^–^32^ MONAP belongs to angiogenesis-related genes. Its overexpression is associated with the pathogenesis and progression of a variety of cancers.^33^–^37^ Our rules imply that gene MONAP is up-regulated in colon tumor samples. It is consistent with the established notion. Moreover, just as Desmin, MONAP is also identified as one of three known hub cancer genes in colon cancer-specific gene network.^28^ hnRNP belongs to the subfamily of ubiquitously expressed heterogeneous nuclear ribonucleoproteins which are associated with pre-mRNAs in the nucleus and appear to influence pre-mRNA processing and other aspects of mRNA metabolism and transport. Thus its dysregulation may cause the occurrence of cancers. Hevin encodes the protein which is implicated in tumor cell growth, differentiation and metastasis, and may play the role of tumor-suppressor.^38^–^44^ Our rules show that if Hevin is down-regulated in the colon tissue samples, then the samples are more likely from the colon tumor patients. It rightly defends the argument that Hevin is the repressor of tumors. EF1R is associated with several functions including translation elongation, actin filament depolymerization, apoptosis, and ubiquitin-mediated protein degradation, etc. Its role in oncogenesis has been investigated by some researchers.^45^–^49^ Calgizzarin encodes the protein which belongs to the group of S100 proteins involved in the Ca^2+^ signaling network, and regulates intracellular activities such as cell growth and motility, cell cycle progression, transcription, and cell differentiation^50^,^51^ Chromosomal rearrangements and altered expression of this gene have been implicated in tumor metastasis. In,^52^ calgizzarin is characterized as a proteomic marker of colorectal cancer due to its significant up-regulation in colorectal carcinoma. The same observation is provided in.^53^–^55^ Tanaka et al detect that the expression of human calgizzarin is remarkably elevated in colorectal cancers compared with that in normal colorectal mucosa by a large scale random cDNA sequencing and Northern blot analysis.^56^ Our rules express the same tendency that calgizzarin is over-expressed in colon tumors. Likewise, our rules demonstrate that TPM1 is down-regulated in colon tumor that coincides with the finding reported in.^57^ Our rules exhibit that PCBD1 is up-regulated in colon tumor, but very few literatures reports the same result. Additionally, there are several genes tightly associated with colon tumor among the marked gene pairs. In our rules, if MIF (macrophage migration inhibitory factor) is up-regulated, then the sample tends to come from tumor tissue. A number of investigations have demonstrated that MIF promotes colon tumor and the other cancers.^58^–^63^ Thus, our rules conform to the documented evidence. CDH3 has been found to be involved in a broad spectrum of cancers including colorectal cancer.^64^–^71^ The gene is identified as accurate prognostic indicator of several tumors due to its marked up-regulation in these tumors.^66^,^68^,^71^,^72^ Our rules show that it is over-expressed in colon tumor as well.

In summary, the majority of important genes relevant to the pathogenesis of colon tumor are marked by our method. The other identified up-regulated genes include Hsp60, Human serine kinase mRNA, IPL1, HYPOTHETICAL PROTEIN IN TRPE 3’REGION and COL11A2 while down-regulated genes encompass MYL9, ACTIN, MaxiK, MGP, GLUT4, MYOSIN HEAVY CHAIN and HCC-1. Some of them have definite biological meaning while the others remain to be explored. Here what we want to emphasize is that the genes distinguishing tumor from normal tissues well involve not only muscle-specific ones but also non-muscle-specific portion. This is in agreement with the finding reported in.^22^ It also reflects the complexity of cancerous pathogenesis.

### Lung cancer dataset

In this dataset, we identify eight genes with comparatively strong prediction power individually. Our rules reveal that the reduced expression of each gene is correlated with the poor prognosis of the cancer. Owing to five out of the eight genes have no annotation available in raw dataset, we only learn about the other three genes: TCEAL1, GEMIN5 and TMPO. TCEAL1, also named as p21, which belongs to the Cip/Kip family of cyclin dependent kinases, has been identified as a gene whose product is tightly associated with development and metastasis of several cancers.^73^–^77^ Direct and indirect evidence has proved that a decrease in the expression levels of the gene might enhance tumor formation, progression and bad prognosis. GEMIN5 encodes the protein which is part of a large macromolecular complex localized to both the cytoplasm and the nucleus that plays a role in the cytoplasmic assembly of small nuclear ribonucleoproteins (snRNPs). In,^78^ Lee et al suggest that Gemin5 overexpression inhibts tumor cell motility so as to may play a role of suppressing metastatic progression. This conforms to our rules. We have not found any evidence indicating that the expression levels of TMPO were correlated with prognosis of cancers. But there are investigations showing that the gene is deregulated in various human tumors.^79^,^80^

In addition, we marked eight gene pairs with good prediction performance. Apart from the non-annotated genes, the involved genes encompass SMAC, PFDN2, FLJ10829, LOC51646, FLJ10326, FLJ12438, GYS10.145 and MSH5. Our rules imply that the decreased expression of these genes indicate a poor prognosis of NSCLC patients- relapse or metastasis. SMAC encodes an inhibitor of apoptosis protein (IAP)-binding protein. A wide variety of investigations have revealed the low expression levels of SMAC correlate with a worse prognosis in many tumor types including NSCLC.^81^–^92^ At the same time, some researchers propose the idea of treating cancers by enhancing SMAC expression in tumor cells.^83^,^85^–^87^,^89^ MSH5 encodes a member of the mutS family of proteins that are involved in DNA mismatch repair or meiotic recombination (MMR) processes. It is a strong candidate for lung cancer susceptibility as deficiency of MMR has been documented to have a role in lung cancer.^93^ Hence, it is quite possible that the downregulation of the gene results to unfavorable clinical outcome of tumors.

### DLBCL dataset

In this dataset, we marked four genes with relatively excellent prediction ability individually. The four genes are EZF, IGF2, CEBPE and BTF1. Our rules indicate that elevated expression of EZF, CEBPE or BTF1 may cause a worse prognosis of DLBCL while abundant expression of IGF2 implies a better prognosis. In,^93^ IGF2 is also identified as a positive indicator of DLBCL prognosis. Whereas previous investigation indicates that these genes are involved in cancerous pathogenesis, further biological insights remain to be clarified.

Some genes lying in the gene pairs we selected in the dataset maybe have important biological relevance. DBP is responsible for high, tissue-specific expression of albumin in fully differentiated hepatocytes, which is expressed by adult not fetal liver cells, and is quickly down-regulated in proliferating hepatocytes.^94^ Our rules indicate that if the gene is down-regulated in one DLBCL patient, then the patient is inclined to have a favorable prognosis. That sounds reasonable. TGM2 encodes the protein which is the enzyme that catalyzes the crosslinking of proteins and appears to be involved in apoptosis. Oudejans et al point out that differences in apoptosis resistance occurring between DLBCL samples link up with distinct clinical outcome.^95^ Since the abundant expression of TGM2 activates the induction of the apoptosis, the upregulation of the gene might mean an excellent prognosis. Our rules reflect the tendency. In addition, in,^96^ Mishra et al suggest that TGM2 modification of p53 oncoprotein could be an additional mechanism whereby TGM2 could facilitate apoptosis. In,^97^ Mangala et al hold that TGM2-induced alterations in the extracellular matrix could effectively inhibit the process of metastasis. In,^98^ Xu et al argue that TGM2 acts as an inhibitor of tumor progression in combination with another gene. PDCD4 encodes a protein localized to the nucleus in proliferating cells which is thought to play a role in apoptosis but the specific role has not yet been determined. Our rules imply that decreased expression of the gene is associated with a good prognosis. It appears to contradict with some previous reports,^99^–^103^ whereas Lankat-Buttgereit et al point out that the function of Pdcd4 might be cell type specific and a role for Pdcd4 in apoptosis or as a tumor suppressor might be limited to certain cell types.^104^ The other identified genes like HRES-1, DTNA,VIPR1, BTF1, HAB1, PTPRN2, EIF2B, IQGAP2 etc., overall possess strong class discriminative power, while their biological mechanism indicating the clinical outcome of DLBCL or other tumors remain unclear.

## Conclusion

Using gene expression patterns to conduct classification or prediction of cancer is often faced with the dilemma: genes (features) far outnumber samples (instances), which will bring about weak prediction efficiency or effect if the model is not chosen reasonably. Another concern is the interpretability of the prediction model when biologists and clinician care for your investigation. Here we employ feature selection to overcome the first difficulty and decision rules to handle the second trouble. We propose one way of feature selection on the basis of the depended degree, a concept from rough set theory. As the canonical definition of the depended degree is too stringent to perform feature selection well, we extend its definition under soft computing consideration. We define the concept of *α* depended degree, whereby we are capable of screening highly discriminative features. Additionally, our work is in accordance with the principle of Occam’s razor: when deciding among many models which make equivalent predictions, choose the simplest one. For this purpose, we only use single genes or gene pairs to build decision rules, which are used to execute prediction of cancer. Results demonstrate that our models work well in that the picked single genes and gene pairs overall give rise to excellent prediction, and meanwhile some biologically significant genes are identified. In general, our method is simpler and more interpretable than most of previously proposed approaches, since our model is based on rules and our rules are created via very few genes. Moreover, our model is robust as we are able to tune our parameters to meet different datasets. Indeed, through comparison, we discover our method outperforms or at least match other algorithms in simplicity and efficacy. It is not strange at all that one or two-gene models are able to result in accurate cancerous prediction because the single genes or gene pairs possibly are the biological or clinical indicators of some specific cancer or general cancer. It appears that one or two gene prediction models are overly simple in that the routine belief is that cancerous pathogenesis is involved in complex systems composed of multi-genes. Whereas our models do not violate the habitual notion in that we have various genes or gene pairs which can cause accurate prediction individually so as to be regarded as candidate markers of cancer. In contrast, some prediction models are not applicable for they contain too many parameters (genes) so that overfitting happens easily. Similar idea is expressed in^4^,^7^,^13^,^15^,^105^ as well. Another advantage of our models is that significant biomarkers can be identified with ease thanks to the operation of few genes once while it is hard to assess which gene is more important by multi-gene models for they run on the basis of a group of genes.

We test our method on several gene expression datasets including CNS tumor, colon tumor, lung cancer and DLBCL. In each dataset, we identify several important genes with documented biological relevance to the malignancy or the cell type. In the CNS tumor dataset, some significant genes like GPS2, beta-NAP, KIAA0220 gene, NSCL1 etc., are identified. In the colon tumor dataset, we succeed in choosing the genes highly related to colon tumor or other tumors. They include Desmin, CRP, MONAP, hnRNP, Hevin, EF1R, calgizzarin, TPM1, PCBD1, MIF etc., wherein calgizzarin has been emphasized as a proteomic marker of colorectal cancer.^52^ In the lung dataset, TCEAL1, GEMIN5, TMPO, SMAC, MSH5 etc. genes associated with the pathogenesis and progression of a variety of cancers are marked by us. In the DLBCL dataset, IGF2, DBP, TGM2, PDCD4 etc., are identified. Their close relationship with tumor occurrence, progression, metastasis and relapse has been widely explored.

Generally speaking, most of the genes associated with tumors encode the proteins involved in cell growth, motility and differentiation, apoptosis, angiogenesis, metabolism, chromosomal rearrangement and translocation, and immune reaction. It is worth noting that whereas there may exist a few particular markers for some specific tumor, a majority of tumor markers might be shared by several tumors. In addition, it is possible that the repressor of some tumor acts as the promoter of another tumor. And it is not impossible that the enhancer of some tumor in one stage transforms into the inhibitor of the same tumor during the other stage.

Another issue concerned with molecular prediction of cancer is whether the prediction performance of one gene or gene set is proportional to its biological interest. We identify some genes which own strong prediction power while their biological or clinical involvements remain unobvious. Whether these genes are indeed correlated to the pathogenesis of cancer, or merely coincidence? This is an important problem, deserving further investigation.

In summary, our method uses very few genes to build rule classifiers of cancer. These classifiers can carry out comparatively accurate prediction. The efficacy of our method has been manifested to be satisfactory by testing on four gene expression datasets. Our follow-up study is to examine our method by more microarray data, including multi-class datasets. In addition, we plan to design more powerful and robust rule classifiers in conjunction with other machine learning algorithms.

## Methods and Materials

### Rough sets

In reality, when we are faced with a heap of data, we often want to learn about them with already known knowledge. However, a majority of data cannot be precisely defined by known knowledge. Thus, in rough set theory, Pawlak describes ill-defined data by designing two concepts: upper approximations and lower approximations, based on the equivalence relation, which is also referred to as one *knowledge* on the studied object set.

**Definition 1** Let *U* be a universe of discourse, *X* ⊆ *U*, and *R* is an equivalence relation on *U. U/R* represents the set of the equivalence class of *U* induced by *R. R* _*_ *X*, R * X, *br*(*R*, *X*), *pos*(*R*, *X*) and *neg*(*R*, *X*) represent the *lower approximation*, *upper approximation*, *boundary region*, *positive region* and *negative region* of *X* on *R* in *U*, respectively, where

$$\begin{array}{l} R_{*}X=\cup \{Y\in U/R|Y\subseteq X\},\\ R^{*}X=\cup \{Y\in U/R|Y\cap X\neq \emptyset \},\\ pos(R,X)=R_{*}X,\\ br(R,X)=R^{*}X-R_{*}X,\\ neg(R,X)=U-R^{*}X. \end{array}$$

If *R* ^*^ *X* = *R* _*_ *X*, then *X* is called *definable* or the *precise set* on *R*; otherwise *X* is called *indefinable* or the *rough set* on *R*.^6^

The data studied by rough set theory are mainly organized in the form of decision tables. One decision table can be represented as *S*= ( *U*, *A* = *C* ∪ *D*), where *U* is the set of samples, *C* the condition attribute set and *D* the decision attribute set. In the decision table, we define the function *I_a_* that maps a member (sample) of *U* to the value of the member on the attribute *a* (*a ∈ A*), and an equivalence relation *R*(*A*’) induced by the attribute subset *A*’ ⊆ *A* as: for *x*, *y ∈ U*, *xR*(*A*’) *y* if and only if *I_a_*(*x*) = *I_a_*(*y*) for each *a ∈ A*’.

In,^17^ Pawlak defines a decision logic language (*DLL*) for decision table *S*= ( *U*, *A* = *C* ∪ *D*) as: each (*a*, *v*) is an atomic formula, where *a* ∈ *A* and *v ∈ V_a_* (set of all the values of a); if ϕ and ψ are formulas, then so are ¬ϕ, ϕ∧ψ, ϕ∨ψ, ϕ→ψ, and ϕ↔ψ. The semantics of *DLL* are defined through the model of decision tables. The *satisfiability* of a formula ϕ by an object *x* in *S*, denoted by *x* ╞ _S_ϕ or for short *x* ╞ ϕ if *S* is understood, is defined by the following conditions:

1. *x* ╞ (*a*, *v*) if and only if *I_a_*(*x*) = *v*,
2. *x* ╞ ¬ϕ if and only if not *x* ╞ ϕ,
3. *x* ╞ ϕ∧ψ if and only if *x* ╞ ϕ and *x* ╞ ψ,
4. *x* ╞ ϕ∨ψ if and only if *x* ╞ ϕ or *x* ╞ ψ,
5. *x* ╞ ϕ→ψ if and only if *x* ╞ ¬ϕ∨ψ,
6. *x* ╞ ϕ↔ψ if and only if *x* ╞ ϕ → ψ and *x* ╞ ψ→ϕ.

We call the set *m_S_*(ϕ) = {*x* ∈ *U* | *x* ╞ *_S_*ϕ} the *meaning* of formula ϕ in decision table *S. m_S_*(ϕ) is simply written as *m*(ϕ) if *S* is understood. On the other hand, we call ϕ a *description* of object set *m*(ϕ). Obviously, the following properties hold:

- *m*((*a*, *v*)) = {*x* ∈ *U* | *I_a_*(*x*) = *v*},
- *m*(¬ϕ) = ∼*m*(ϕ),
- *m*(ϕ∧ψ) = *m*(ϕ) ∩ *m*(ψ),
- *m*(ϕ∨ψ) = *m*(ϕ) ∪ *m*(ψ),
- *m*(ϕ→ψ) = ∼*m*(ϕ) ∪ *m*(ψ),
- *m*(ϕ↔ψ) = (*m*(ϕ) ∩ *m*(ψ)) ∪ (∼*m*(ϕ) ∩ ∼*m*(ψ)).

In rough set theory, the *depended degree* of an attribute subset *P* by an attribute subset *Q* is denoted by γ *_p_*(*Q)* and is defined as

$$\gamma_{P}(Q)=\frac{\left|{\text{POS}}_{P}(Q)\right|}{\left|U\right|},$$

where $\left|{\text{POS}}_{P}(Q)|=|\underset{X\in U/R(Q)}{\cup }pos(P,X)\right|$ represents the size of the union of the positive region of each equivalence class in *U/R*(*Q*) on *P* in *U*, and *U* represents the size of *U* (set of samples).

If *Q* is the decision attribute *D*, and *P* a subset of condition attributes, then γ *_p_*(*D*) indicates the depended degree of the condition attribute subset *P* by the decision attribute *D*. It means that, to what degree, *P* can discriminate the distinct classes of *D*. Thus, γ *_p_*(*D*) rightly reflects the classification power of the subset *P* of condition attributes. The greater γ *_p_*(*D*) is, the stronger classification ability *P* inclines to possess.

Rough set theory tries to discover the simplest *decision rules* with the equivalent explaining power and classification performance as more complicated rules. One decision rule with the form of “*A* ⇒ *B*” indicates that “if *A*, then *B*”, where *A* is the description of condition attributes and *B* the description of decision attributes. The *confidence* of a decision rule *A* ⇒ *B* is defined as:

$$\text{confidence }(A\Rightarrow B)=\frac{\text{support }(A\wedge B)}{\text{support }(A)},$$

where support (*A*) denotes the proportion of the samples satisfying *A* and support (*A* ∧ *B*) the proportion of the samples satisfying *A* and *B* simultaneously. According to the *DLL*, the *confidence* of a decision rule *A* ⇒ *B* is rewritten as:

$$\text{confidence }(A\Rightarrow B)=\frac{\left|m(A)\cap m(B)\right|}{\left|m(A)\right|}.$$

The confidence of a decision rule implies the reliable degree of the rule. If one decision rule has 100% confidence, we call it the *consistent decision rule*.

In the previous studies of classifying cancer by gene expression profiles using rough set theory, the measure of depended degree is often set as the basis of ranking genes.^9^,^10^ However, as the canonical definition of depended degree is overly stringent, sometimes it is not able to rightly express the discriminatory power of features. Hence, here we extend the definition of depended degree under soft computing consideration.

**Definition 2** Let *U* be a universe of discourse, *X* ⊆ *U*, 0 ≤ *α* ≤ 1 and *R* is an equivalence relation on *U. pos*(*R*, *X*, *α*) representing the *α positive region* of *X* on *R* in *U*, is defined as:

pos(R,X,α)=∪{Y∈U/R||Y∩X|/|Y|≥α}.

Correspondingly, the *α depended degree* of an attribute subset *P* by an attribute subset *Q*, denoted by γ *_p_*(*Q*, *α*), is defined as:

$$\gamma_{P}(Q,\alpha )=\frac{\left|{\text{POS}}_{P}(Q,\alpha )\right|}{\left|U\right|}$$

where $\left|{\text{POS}}_{P}(Q,\alpha )|=|\underset{X\in U/R(Q)}{\cup }pos(P,X,\alpha )\right|$ represents the size of the union of the *α positive region* of each equivalence class in *U*/*R* (*Q*) on *P* in *U*.

Obviously, the definition of *α* depended degree is a generalization of the definition of depended degree as when *α* equals to 1, both definitions are equivalent. We choose *α* depended degree instead of depended degree as the basis of screening features. Once *α* value is determined, we only choose the genes or gene pairs with 1 of γ *_p_*(*D*, *α*) value to build classification (decision) rules. Suppose g is one of the selected genes and *U* sample set. *U/R*(*g*) = {*c*_1_(*g*), *c*_2_(*g*), …, *c_n_*(*g*)} represents the set of the equivalence class of samples induced by *R*(*g*). Two samples *s*_1_ and *s*_2_ belong to the same equivalence class of *U/R*(*g*) if and only if they have the same value on *g*. In addition, we represent the set of the equivalence class of samples induced by *R*(*D*) as *U/R*(*D*) = {*d*_1_(*D*), *d*_2_(*D*), …, *d_m_*(*D*)}, where *D* is the class (decision) attribute. Two samples *s*_1_ and *s*_2_ belong to the same equivalence class of *U/ R*(*D*) if and only if they have the same value on *D*. For each *c*_i_(*g*) (*i* = 1, 2, …, *n*), if there exists some *d_j_*(*D*) ( *j* ∈ {1, 2,…, *m*}), satisfying |*c_i_*(*g*) ∩ *d_j_*(*D*)|/ |c*_i_*(*g*)| ≥ *α*, then we generate the classification rule: *A*(*c_i_*(*g*)) ⇒ *B*(*d_j_*(*D*)), where *A*(*c_i_*(*g*)) is the formula describing the sample set *c_i_*(*g*) by *g* value and *B*(*d_j_*(*D*)) is the formula describing the sample set *d_j_*(*D*) by the class value. In the case of gene pairs, we construct classification rules through the same strategy. Here what we want to emphasize is that only the single genes or gene pairs chosen by all the leave-one-out training sets are used for building classification rules.

The confidences of the rules generated by our approach depend on *α*. The following theorem states the relationship between *α* and the confidences of the induced rules.

**Theorem 1** The confidence of each induced decision rule by our way is no less than *α*.

Proof. For any condition attribute subset *P* of size one or two, if γ *_p_*(*D*, *α*) = 1, then *P* will be chosen by our way. Suppose the decision rule *A* ⇒ *B* is produced by *P*. Then by our way, we have *m*(*A*) ∈ *U/R*(*P*), *m*(*B*) ∈ *U/R*(*D*) and |*m*(*A*) ∩ *m*(*B*)|/|*m*(*A*)| ≥ *α*. As confidence ( *A*⇒ *B*) = |*m*( *A*) ∩ *m*( *B*)| / | *m*( *A*)| , the conclusion is founded.

Therefore, by tuning *α* value, we can not only control the size of the set of selected single genes or gene pairs, but also ensure the confidence of derived decision rules.

For the cancer classification problem, every microarray data collected can be represented as a decision table with the form of Table 13. In the microarray data decision table, there are *m* samples and *n* genes. Every sample is assigned to one class label. *g*(*x*, *y*) represents the expression level of gene *y* in sample *x*.

### Dataset

#### CNS tumor dataset

The dataset is about patient outcome prediction for central nervous system embryonal tumor.^19^ In this dataset, there are 60 observations, each of which is described by the gene expression levels of 7129 genes and a class attribute with two distinct labels—Class 1 (survivors) versus Class 0 (failures). Survivors are patients who are alive after treatment while the failures are those who succumbed to their disease. Among 60 patient samples, 21 are labeled as “Class 1” and 39 are labeled as “Class 0”.

#### Colon tumor dataset

The dataset contains 62 samples collected from colon-cancer patients.^22^ Among them, 40 tumor biopsies are from tumors (labeled as “negative”) and 22 normal (labeled as “positive”) biopsies are from healthy parts of the colons of the same patients. Each sample is described by 2000 genes.

#### Lung cancer dataset

The dataset contains 39 NSCLC (Non-Small Cell Lung Cancer) samples, 24 of which are from patients with metastasis (labeled as “relapse”) and 15 are from the patients with disease-free based on both clinical and radiological testing (labeled as “non-relapse”).^23^ The total number of genes is 2880.

#### DLBCL dataset

The dataset is about patient outcome prediction for DLBCL.^24^ The total of 58 DLBCL samples are from 32 cured patients (labeled as ‘cured’) and 26 refractory patients (labeled as ‘fatal’). The gene expression profile contains 6817 genes.

Table 14 summarizes the four gene expression datasets.

### Data preprocessing

As there exist a few missing attribute values in the lung cancer dataset, we first fill each of them with the mean of all the attribute values from the same class of samples as the sample containing the missing value.

Because rough set theory is suitable for handling discrete attributes, we discretize all the training set decision tables. We utilize the entropy-based discretization method, proposed by Fayyad et al.^21^ This algorithm recursively applies an entropy minimization heuristic to discretize the continuous-valued attributes. The stop of the recursive step for this algorithm depends on the minimum description length (MDL) principle. We implement the discretization in the Weka package.^106^ Every continuous-valued attribute is discretized into a one-category, two-category or three-category attribute. Table 15 shows the discretized decision table for the CNS tumor with the first sample left out. We execute our algorithm for the feature selection and decision rule induction using this kind of tables.

### Validation

We employ leave-one-out cross-validation approach. For the dataset containing *n* samples, each sample is left out in turn, and the learning algorithm is trained on all the remaining *n-1* samples. Then the training result is tested on the left-out sample. The final estimate is the average of *n* test results.

## Figures and Tables

**Table 1:** 6 genes with high prediction accuracy in the CNS tumor dataset.

Probe ID	Correctly-classified sample number (accuracy)	α
U28963_at	47 (78%)	0.75
X99050_rna1_at	45 (75%)	0.75
D83542_at	46 (77%)	0.7
S71824_at	50 (83%)	0.7
U37673_at	40 (67%)	0.7
D86974_at	45 (75%)	0.7

**Table 2:** 11 gene pairs with high prediction accuracy in the CNS tumor dataset.

1st – 2nd Probe ID	Correctly-classified sample number (accuracy)	α
D83542_at–S71824_at	54 (90%)	0.85
D31763_at–U08998_at	54 (90%)	0.8
D83542_at–X99050_rna1_at	49 (82%)	0.8
D83542_at–D86974_at	52 (87%)	0.8
L33243_at–U36448_at	52 (87%)	0.8
M73547_at–U74324_at	51 (85%)	0.8
M96739_at–U36448_at	54 (90%)	0.8
S71824_at–D86974_at	51 (85%)	0.8
U37143_at–D43682_s_at	48 (80%)	0.8
U79277_at–D43682_s_at	47 (78%)	0.8
X99050_rna1_at–D86974_at	49 (82%)	0.8

**Table 3:** 21 genes with high prediction accuracy in the colon tumor dataset.

GenBank accession no.	Correctly-classified sample number (accuracy)	α
M63391	52 (84%)	0.8
M76378	50 (81%)	0.8
J02854	50 (81%)	0.8
M26383	52 (84%)	0.8
M76378	50 (81%)	0.75
T60155	48 (77%)	0.75
M22382	50 (81%)	0.75
X12671	49 (79%)	0.75
M76378	50 (81%)	0.75
T96873	47 (76%)	0.75
X86693	47 (76%)	0.75
J05032	50 (81%)	0.75
U25138	48 (77%)	0.75
T60778	47 (76%)	0.75
M91463	48 (77%)	0.75
R87126	51 (82%)	0.7
T51571	46 (74%)	0.7
T92451	48 (77%)	0.7
U09564	48 (77%)	0.7
R97912	45 (73%)	0.7
L41559	45 (73%)	0.7

**Table 4:** 16 gene pairs with high prediction accuracy in the colon tumor-dataset.

1st – 2nd GenBank accession no.	Correctly-classified sample number (accuracy)	α
T51571–J02854	56 (90%)	0.9
J02854–L41559	56 (90%)	0.9
M76378–M63391	52 (84%)	0.85
M63391–M76378	52 (84%)	0.85
M63391–Z49269	45 (73%)	0.85
M63391–X86693	53 (85%)	0.85
Z50753–H40095	55 (89%)	0.85
R87126–H81068	55 (89%)	0.85
X12671–J02854	56 (90%)	0.85
X12671–M26383	54 (87%)	0.85
M76378–M26383	55 (89%)	0.85
H40095–M36634	54 (87%)	0.85
R97912–J02854	55 (89%)	0.85
R97912–M26383	54 (87%)	0.85
R06601–X63629	54 (87%)	0.85
M36634–H08393	56 (90%)	0.85

**Table 5:** 8 genes with high prediction accuracy in the lung cancer dataset.

Unigene ID	Correctly-classified sample number (accuracy)	α
505266^a^	32 (82%)	0.75
Hs.95243	32 (82%)	0.75
Hs.25882	32 (82%)	0.75
Hs.275198	32 (82%)	0.75
36491^a^	32 (82%)	0.75
Hs.170225	33 (85%)	0.75
Hs.17258	29 (74%)	0.75
Hs.11556	31 (79%)	0.75

a The Unigene ID is not available.

**Table 6:** 8 gene pairs with high prediction accuracy in the lung cancer dataset.

1st – 2nd Unigene ID	Correctly-classified sample number (accuracy)	α
Hs.169611–Hs.285701	31 (79%)	0.75
Hs.285701–Hs.132415	29 (74%)	0.75
Hs.285701–Hs.57655	30 (77%)	0.75
Hs.57655–Hs.8595	31 (79%)	0.75
Hs.184542–Hs.58323	31 (79%)	0.75
Hs.262823–Hs.8595	31 (79%)	0.75
Hs.262480–Hs.772	32 (82%)	0.75
Hs.112193–505266^a^	31 (79%)	0.75

**Table 7:** 4 genes with high prediction accuracy in the DLBCL dataset.

Probe ID	Correctly-classified sample number (accuracy)	α
U70663_at	44 (76%)	0.7
M17863_s_at	44 (76%)	0.7
U48865_s_at	43 (74%)	0.7
U90543_at	45 (78%)	0.7

**Table 8:** 20 gene pairs with high prediction accuracy in the DLBCL dataset.

1st – 2nd Probe ID	Correctly-classified sample number (accuracy)	α
AFFX-BioC-3_at–M95925_at	46 (79%)	0.8
AFFX-BioC-3_at–U70663_at	48 (83%)	0.8
AFFX-M27830_5_at – X70811_at	49 (84%)	0.8
AFFX-M27830_5_at – U46744_at	49 (84%)	0.8
AC002450_at–M95925_at	47 (81%)	0.8
AC002450_at–U48213_at	47 (81%)	0.8
AC002450_at–HG4020-HT4290_s_at	48 (83%)	0.8
M95925_at–X70811_at	46 (79%)	0.8
U23028_at–U70663_at	47 (81%)	0.8
U23028_at–X70811_at	48 (83%)	0.8
U51903_at–U70663_at	48 (83%)	0.8
U51903_at–X70811_at	47 (81%)	0.8
U66702_at–U70663_at	47 (81%)	0.8
U66702_at–HG4020-HT4290_s_at	48 (83%)	0.8
U66702_at–U90543_at	52 (90%)	0.8
U70663_at–U83908_at	47 (81%)	0.8
U70663_at–X83412_at	46 (79%)	0.8
U70663_at–X77777_s_at	47 (81%)	0.8
U70663_at–X16660_cds1_s_at	46 (79%)	0.8
U70663_at–U46744_at	47 (81%)	0.8

**Table 9:** Comparison of best prediction accuracy for the CNS tumor dataset.

Methods (feature selection + classification)^b^	# Selected genes	# Correctly-classified samples (accuracy)
*α* depended degree + decision rules	1	50 (83%)
[this work]	2	54 (90%)
Signal to noise ratios + k-NNs^19^	8	47 (78%)
Signal to noise ratios + Weighted voting^19^	1–200	46 (77%)
Signal to noise ratios + SVMs^19^	150	45 (75%)
Signal to noise ratios + SPLASH^19^	1–200	45 (75%)
Signal to noise ratios + TrkC^19^	1	40 (67%)
Signal to noise ratios + Staging^19^	1–200	41 (68%)
Signal to noise ratios + staging, k-NNs and TrkC^19^	1–200	48 (80%)
Signal to noise ratios + SVM, k-NNs and TrkC^19^	1–200	48 (80%)
HFW + C4.5^20^	20	45 (75%)
HFW + NaiveBayes^20^	29	52 (86.67%)
Discretization + Single C4.5^12^	74^c^	51 (85%)^d^
Discretization + Bagging C4.5^12^	74^c^	53 (88%)^d^
Discretization + AdaBoost C4.5^12^	74^c^	53 (88%)^d^

b The methods include two sections: feature selection methods and classification methods. The decision trees classification methods are also involved in feature selection.

c 74 is the number of the genes withheld for the actual learning process instead of the number of the genes contained in the decision trees, which is not provided in.^12^

d Tenfold cross-validation accuracy is provided.

**Table 10:** Comparison of best prediction accuracy for the colon tumor dataset.

Methods (feature selection + classification)	# Selected genes	# Correctly-classified samples (accuracy)
*α* depended degree + decision rules	1	52 (84%)
[this work]	2	56 (90%)
HykGene + k-NNs, SVMs, C4.5, NB^107^	3	57 (92%)
MAVE + logistic discrimination^108^	50	52 (84%)
Clustering and rough sets attribute reduction + k-NNs^109^	6	49 (79%)
Clustering and rough sets attribute reduction + NB^109^	6	51 (82%)
Clustering and rough sets attribute reduction + C5.0^109^	6	56 (90%)
MRMR + NB^110^	9	58 (94%)
RBF + C4.5^111^	4	58 (94%)
ReliefF + C4.5^111^	4	53 (85%)
CFS-SF + C4.5^111^	26	55 (89%)

**Table 11:** Comparison of best prediction accuracy for the lung cancer dataset.

Methods (feature selection + classification)	# Selected genes	# Correctly-classified samples (accuracy)
*α* depended degree + decision rules	1	33 (85%)
[this work]	2	32 (82%)
HFW + C4.5^20^	12	35 (90%)
HFW + NaiveBayes^20^	18	35 (90%)
FCBF + C4.5^20^	12	31 (79%)
FCBF + NaiveBayes^20^	12	24 (62%)
CFS-SF + C4.5^20^	13	26 (67%)
CFS-SF + NaiveBayes^20^	13	24 (62%)
ReliefF + C4.5^20^	12	24 (62%)
ReliefF + NaiveBayes^20^	18	25 (64%)

**Table 12:** Comparison of best prediction accuracy for the DLBCL dataset.

Methods (feature selection + classification)	# Selected genes	# Correctly-classified samples (accuracy)
α depended degree + decision rules	1	48 (78%)
[this work]	2	52 (90%)
Signal to noise ratios + Weighted voting^24^	13	44 (76%)
Signal to noise ratios + k-NNs^24^	9	41 (71%)
Gradient descent algorithm + SVMs^24^	unknown^e^	45 (78%)
HFW + C4.5^20^	22	44 (76%)
HFW + NaiveBayes^20^	19	50 (86%)
FCBF + C4.5^20^	27	27 (47%)
FCBF + NaiveBayes^20^	27	31 (53%)
ReliefF + C4.5^20^	22	25 (43%)
ReliefF + NaiveBayes^20^	19	31 (53%)

e No related data is provided.

**Table 13:** Microarray data decision table.

Samples	Condition attributes (genes)				Decision attributes (classes)
	Gene 1	Gene 2	…	Gene *n*	Class label
1	*g* (1, 1)	*g* (1, 2)	*…*	*g* (1, *n*)	*Class* (1)
2	*g* (2, 1)	*g* (2, 2)	*…*	*g* (2, *n*)	*Class* (2)
…	*…*	*…*	*…*	*…*	*…*
…	*…*	*…*	*…*	*…*	*…*
*m*	*g* (*m*, 1)	*g* (*m*, 2)	*…*	*g* (*m, n*)	*Class* (*m*)

**Table 14:** Summary of the four gene expression datasets.

Dataset	# Original genes	Class	# Samples
CNS Tumor	7129	Class 1/Class 0	60 (21/39)
Colon Tumor	2000	negative/positive	62 (40/22)
Lung Cancer	2880	relapse/non-relapse	39 (24/15)
DLBCL	6817	cured/fatal	58 (32/26)

**Table 15:** Discretized CNS tumor decision table with the first sample left out.

Samples	Condition attributes (genes)^f^							Decision attributes (classes)
	Gene 1	…	Gene 11	…	Gene 18	…	Gene 7129	Class label
1	‘All’	…	‘(-inf-187]’	…	‘(−330-inf]’	…	‘All’	Class 1
2	‘All’	…	‘(-inf-187]’	…	‘(−330-inf]’	…	‘All’	Class 1
…	…	…	…	…	…	…	…	…
20	‘All’	…	‘(-inf-187]’	…	‘(−330-inf]’	…	‘All’	Class 1
21	‘All’	…	‘(-inf-187]’	…	‘(−330-inf]’	…	‘All’	Class 0
22	‘All’	…	‘(187-inf]’	…	‘(−330-inf]’	…	‘All’	Class 0
…	…	…	…	…	…	…	…	…
58	‘All’	…	‘(-inf-187]’	…	‘(-inf−330]’	…	‘All’	Class 0
59	‘All’	…	‘(-inf-187]’	…	‘(−330-inf]’	…	‘All’	Class 0

f ‘All’ represents that one gene has the same value in all samples; ‘(-inf-x]’ represents ‘≤x’; ‘(x-inf]’ represents ‘≤x’.
